# Impossible Airway Requiring Venovenous Bypass for Tracheostomy

**DOI:** 10.1155/2012/592198

**Published:** 2012-08-16

**Authors:** Johnathan Gardes, Tracey Straker

**Affiliations:** Department of Anesthesiology, Montefiore Medical Center, Bronx, New York City, NY 10467, USA

## Abstract

The elective surgical airway is the definitive management for a tracheal stenotic lesion that is not a candidate for tracheal resection, or who has failed multiple-tracheal dilations. This case report details the management of a patient who has failed an elective awake tracheostomy secondary to the inability to be intubated as well as severe scar tissue at the surgical site. A combination of regional anesthesia and venovenous bypass is used to facilitate the surgical airway management of this patient. Cerebral oximetry and a multidisciplinary team approach aid in early detection of an oxygenation issue, as well as the emergent intervention that preserved this patient's life.

## 1. Introduction

Proper management of the difficult airway presents one of the most important skill sets for the anesthesiologist to master. However, certain situations necessitate one look beyond traditional algorithms. In this case, a multidisciplinary team of otorhinolaryngologists, cardiac surgeons, perfusionists, and anesthesiologists decided to use venovenous bypass as a means to oxygenate a patient whose airway could not be secured because of severe tracheal stenosis.

## 2. Case Report

**Figure 1:**
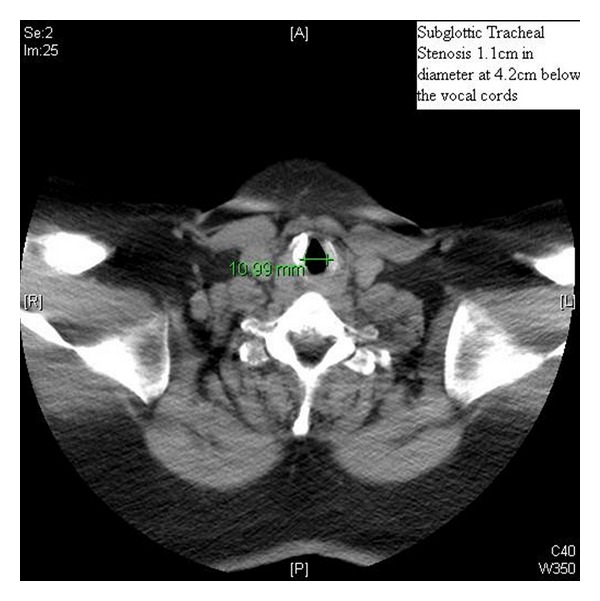
CT SCAN of subglottic tracheal stenosis.

**Figure 2:**
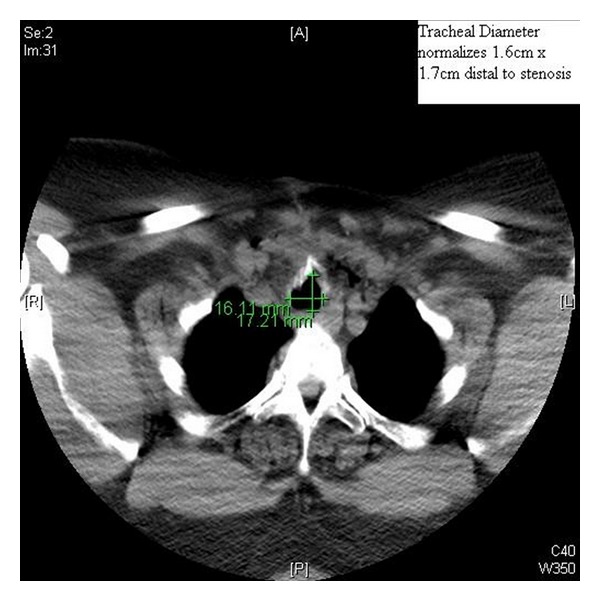
CT SCAN of tracheal diameter.

A 45-year-old woman with a long history of tracheal stenosis and upper airway obstruction presented for elective tracheostomy placement in the setting of supra- and infraglottic stenosis after failed awake tracheostomy by an otorhinolaryngologist (ORL) (Figures 1 and 2). It was felt by the attending ORL surgeon that the airway could not be secured from above after serial diagnostic scopes. Due to the failed awake tracheostomy, it was felt that surgical airway under bypass was the only option.

 Eight years previously, the patient presented for an elective bilateral tubal ligation. At the time, she was otherwise healthy. The intraoperative course was unremarkable and the patient's trachea was extubated on the operating table. However, after moving to the stretcher for transport, she developed acute respiratory distress. She was quickly returned to the operating room (OR) table and her trachea reintubated after several attempts at direct laryngoscopy. At the time, her airway was noted to be acutely swollen and edematous. She remained intubated in the intensive care unit for two weeks during which time several attempts at extubation failed. Finally, she was weaned off support and discharged home. The presumptive diagnosis was an acute allergic reaction to the antibiotic cephazolamine, which she had received intraoperatively.

 Two days after discharge the patient returned to the emergency room in acute respiratory distress and could not be intubated. An emergency tracheostomy was performed to secure her airway. The patient remained with the tracheostomy for the next 7 months before she was successfully decannulated. Since that time, the patient has returned to the operating room several times with tracheal stenosis requiring dilation. Her past medical history was also significant for hypothyroidism, morbid obesity, obstructive sleep apnea requiring continuous positive airway pressure (CPAP), and hypertension. Medications included levothyroxine sodium, furoate monohydrate by nasal inhalation and esomeprazole. Allergies to latex and cephalosporins were reported. Review of symptoms was significant for chronic shortness of breath and three pillow orthopnea. The patient had fasted for more than 8 hours. On physical examination, the patient was mildly hypertensive, with a class III airway, mild stridor, and oozing from the site of the prior tracheostomy. All laboratory values and the cardiogram were within normal limits.

Because of the inability to secure the airway, even by tracheostomy, and repeated incidents of desaturation during prior attempts, the team felt that adequate oxygenation could best be managed by venovenous bypass. Venovenous bypass was suggested after consultation with the cardiac anesthesiology team because oxygenation would be maintained and the cardiac function of the patient was normal. The ORL surgeon requested that we do not cannulate any structures in the neck as they would be working in that area. With this request from the ORL service and the fact that arterial venous bypass provides greater control over hemodynamics, femoral bypass was the chosen area for cannulation.

The anesthetic plan for spinal without sedation was discussed with the patient. The patient was brought into the OR where standard ASA monitors were placed including arterial cannulation and cerebral oximetry. Spinal anesthesia was placed at L4-5 using 24G Gertie Marx spinal needle and 1.2 mL of hyperbaric 7.5% bupivacaine. The patient had a T10 level at 5 minutes with vital signs stable throughout. Of note, the patient started to receive an infusion of vancomycin prior to incision and immediately experienced pruritus and scratchy throat. The antibiotic infusion was promptly discontinued and the patient was given steroids and subcutaneous epinephrine. The symptoms resolved and surgery continued. The patient was heparinized. Just prior to going on femoral venovenous bypass, general anesthesia was induced using midazolam, fentanyl, and etomidate. Once on bypass, vecuronium was given. As the patient was to have a tracheostomy on bypass, general anesthesia was instituted to assure no issues of recall.

Shortly bypass was initiated, cerebral oximetry and pulse oximetry values as well as blood pressure all dropped rapidly, likely from poor brain and upper extremity perfusion caused by the bypass. During cutdown on the patient's vasculature, it was discovered that he had severe tortuosity of the vessels. The cut-down time was extensive and the spinal anesthetic began to wear off. It was thought that one of the differentials for failure of bypass was the creation of a false lumen from the cannulation.

 At this point, the otorhinolaryngologist rapidly secured the airway using rigid laryngoscopy and placed an oral endotracheal tube. With proper ventilation, the vital signs all returned to normal and she was taken off bypass and the heparin was reversed. The tracheostomy was then performed by the surgeon under general anesthesia. The remainder of the intraoperative course was unremarkable and the patient was extubated at the end of the case. The patient did well postoperatively and was discharged home.

## 3. Discussion

The difficult airway algorithm from the American Society of Anesthesiologists details a complex decision tree of basic and advanced airway management choices in both the awake and anesthetized patient who has suspected or known difficult ventilation and/or intubation [1]. The final steps in this algorithm end with invasive or surgical airway access. In this case, the patient had already failed awake surgical airway and was believed to have additional upper airway obstruction and anatomy that would prevent safe intubation either awake or asleep. With the failure of what is usually the final step in the algorithm, the anesthesia team in consultation with other surgical specialists opted for venovenous bypass as a means to oxygenate and anesthetize a patient whose airway could not be secured. While extracorporeal membrane oxygenation (ECMO) has been utilized and reported in the pediatric population fairly extensively, venovenous bypass has only rarely been reported in the literature as a means of oxygenation in the setting of the impossible airway in an adult, often in the setting of a large thyroid or mediastinal tumor or severe tracheal trauma [2, 3]. Rosa and colleagues and Jeon and colleagues both presented cases of cardiopulmonary bypass being safely utilized in this manner for cases of a cervical and thyroid tumor, respectively [4, 5]. Shiraishi and colleagues presented a series of 18 cases of tracheal resection and reconstruction, one of which required percutaneous cardiopulmonary support under minimal sedation [6]. Cardiopulmonary bypass is not without risk and complications as demonstrated by the fall in cerebral oximetry and pulse oximetry in our patient. [7].

The ORL surgeon admitted that this case was transferred to him from a colleague, and he had actually never instrumented the patient's airway in the operating room for a direct laryngoscopy. Perhaps had the otorhinolaryngologist actually instrumented the airway previously, better objective data would have been available to avoid the case outcome.

The otorhinolaryngologist was present throughout the procedure and was able to provide a rapid and safe emergency airway intervention that allowed the patient to be adequately oxygenated. While our patient had an uncomplicated postoperative course, the risk of hemorrhage in this population is significant.

 Even though the patient was unable to tolerate bypass for the length of the tracheostomy procedure and required alternative means of securing the airway, the presence of the multidisciplinary team provided safe and effective backup. We believe that rare and complex airway situations such as this case should always be approached with a multidisciplinary team of surgeons and anesthesiologists as the alternatives to traditional airway management such as venovenous bypass can be extremely challenging and unpredictable.

## References

[1] American Society of Anesthesiologists Task Force on Management of the Difficult Airway (2003). Practice guidelines for management of the difficult airway: an updated report by the American Society of Anesthesiologists task force on management of the difficult airway. *Anesthesiology*.

[2] Raake J, Johnson B, Seger B (2011). Extracorporeal membrane oxygenation, extubation, and lung-recruitment maneuvers as rescue therapy in a patient with tracheal dehiscence following slide tracheoplasty. *Respiratory care*.

[3] SenDasgupta C, Sengupta G, Ghosh K, Munshi A, Goswami A (2010). Femoro-femoral cardiopulmonary bypass for the resection of an anterior mediastinal mass. *Indian Journal of Anaesthesia*.

[4] Rosa P, Johnson EA, Barcia PJ (1996). The impossible airway: a plan. *Chest*.

[5] Jeon H-K, So YK, Yang JH, Jeong HS (2009). Extracorporeal oxygenation support for curative surgery in a patient with papillary thyroid carcinoma invading the trachea. *Journal of Laryngology and Otology*.

[6] Shiraishi T, Yanagisawa J, Higuchi T (2011). Tracheal resection for malignant and benign diseases: surgical results and perioperative considerations. *Surgery Today*.

[7] Belmont MJ, Wax MK, DeSouza FN (1998). The difficult airway: cardiopulmonary bypass—the ultimate solution. *Head and Neck*.

